# Current News and Opinion

**Published:** 1885-12

**Authors:** 


### Current News and Opinion.

### OBITUARY.

DIED.—In Hartford, Conn., Nov. 11th, Dr. John M. Riggs.

Dr. Riggs was born in Seymour, Conn., Oct. 25th, 1810. He graduated at Trinity College, Hartford, in 1837, and soon after commenced the studies which were to fit him for the practice of dentistry. His operations upon human teeth gave evidences of remarkable skill and good judgment, and his fillings of soft gold and tin foil withstood the wear and tear to which they were subjected for several decades of years, with scarcely a perceptible change of appearance.

The first surgical operation ever attempted under the effect of an anæsthetic was performed by Dr. Riggs. His subject was Dr. Horace Wells, also of Hartford, whose office was in an adjoining room of the same building with Dr. Riggs. At a comic exhibition of "laughing gas," given by Mr. C. G. Colton, in Hartford, the evening before, Dr. Wells observed that a young man who had just recovered from the exhilarating effects of the gas had so abraded the skin on his hand as to cause the blood to flow somewhat freely, yet was not conscious of the injury until his attention was directed to the wound by Dr. Wells. Replies from several questions put to the youth deeply impressed Dr. Wells with the belief that "laughing gas" might be of value in surgical cases, by rendering patients insensible to pain. To satisfy himself on this point he determined to give the gas a practical test, and having a superior molar in his own mouth, which at times had given him slight annoyance, he decided to inhale the gas and have the tooth extracted. Mr. Colton was engaged to administer the gas, and when Dr. W. was fully under its influence Dr. Riggs applied the forceps and removed the molar. The experiment proved a perfect success. "Anæsthesia" was thus discovered, and to the world was given one of its greatest blessings. This was on the 11th of December, 1844, which antedates the use of ether as an anæsthetic for nearly two years. After this the nitrous oxide gas was used by both Drs. Wells and Riggs, for several years.

Dr. Riggs achieved quite a reputation in the professional world in his arduous efforts towards learning the nature and arresting the progress of the disease called *pyorrhœa alveolaris*, sometimes known as "Riggs' disease." Probably no other man ever gave to this troublesome malady more thought or greater attention than did Dr. Riggs. As a member of our profession he won an honorable position, and was much esteemed by all who knew him. C. E. F.

---

### CIRCULAR LETTER TO MEMBERS OF SECTION VII. "PHYSIOLOGY AND ETIOLOGY," OF THE AMERICAN DENTAL ASSOCIATION.

*Dear Doctor*—In order to make such a showing of work as our Section should do at the next annual meeting of the Association, it is deemed best to proceed at once to the assignment of subjects, in order to give time for investigation.

At the last meeting of the Section a report was adopted and presented to the Association, saying that we would investigate the following subjects, and report on the same at the next meeting.

(1) Investigation of the Normal and Abnormal Conditions of the Oral Secretions.

(2) The Tongue in Health and Disease.

(3) Personal Hygiene for the Dentist.

(4) Continuation of the Study of the Etiology of Dental Caries.

What we wish now is for you to select one or more of the above subjects, or special parts of these subjects, and prosecute investigation, *as original and new as possible,* in the department you may select. What we need is something *fresh,* even if inconsistent with cherished theories and opposed to the accepted teachings. We wish to progress. We wish to take a step forward, and to that end we must make new discoveries. What will *you* undertake to do by the the next meeting?

If, however, you have anything else on hand which you are working upon, and wish to report it, that will be fully as acceptable as any of the above subjects. Please notify the Secretary, as soon as possible, of the work you intend to do and to report upon, that he may arrange the same in the schedule for the next report of the Section, and that he may assist you as much as possible.

You are aware that, at the last meeting of the Association, an appropriation of two hundred dollars was made to this Section for its use in the prosecution of *original* investigation. You are entitled to a share of this amount for the defraying of expenses you may incur in the prosecution of such investigations. *Provided,* however, that the results of that investigation, so assisted by this fund, shall not be given to any other society nor to any journal before the next meeting of the Association, but shall be first given to the world at that meeting.

Please take a part, be it ever so little, in the work of the Section, and notify the Secretary as soon as possible of your selection. Each must bear his part for the good of the Section and the advancement of knowledge.

H. A. SMITH, Chairman,

A. H. THOMPSON, Secretary.

Topeka, Kan.

### CONNECTICUT VALLEY DENTAL SOCIETY.

At the annual meeting of the Connecticut Valley Dental Society, held in Springfield, Mass., Nov. 5 and 6, the following officers were elected for the ensuing year:

*President*—Dr. E. A. Stebbins, Shelburne Falls, Mass.

*First Vice President*—Dr. I. N. Davenport, Northampton, Mass.

*Second Vice-Presedent*—Dr. F. W. Williams, Greenfield, Mass.

*Secretary*—Dr. Geo. A. Maxfield, Holyoke, Mass.

*Assistant Secretary*—Dr. A. J. Nims, Turner's Falls, Mass.

*Treasurer*—Dr. W. H. Jones, Northampton, Mass.

*Executive Committee*—Dr. L. C. Taylor, Hartford, Conn.; Dr. J. P. Parker, Bellows Falls, Vt.; Dr. W. F. Andrews, Springfield, Mass.

GEO. A. MAXFIELD, Secretary.

---

### A GOOD INVESTMENT.

Two cents a day set aside from ordinary expenses will pay for *three* of the leading dental journals published. This small sum, if thus invested, will yield the best sort of interest by furnishing to subscribers much valuable reading matter, that cannot fail to greatly benefit them in the practice of their specialty. Two cents a day in this way expended would hardly be missed from even the most scanty income—and who does not daily part with a much larger sum for a less worthy purpose? In the many complimentary letters received, referring to the INDEPENDENT PRACTITIONER, more than once has it been asserted that a single suggestion found in the journal has amply paid for a year's subscription.

Let it be borne in mind that "the professional man who does not read the literature of his calling, will always have place in the rear of his profession." C. E. F.

---

### A TREATISE ON DIPHTHERIA.

J. H. Chambers & Co., of St. Louis, the well-known medical publishers, have in press a work on Diphtheria and Croup, by Dr. A. Sanné, of Paris, translated and annotated by Henry Z. Gill, A. M., M. D., LL. D. It will be handsomely illustrated, and will, no doubt, meet with a large sale.

### AMERICAN ACADEMY OF DENTAL SCIENCE.

At the 18th annual meeting of the American Academy of Dental Science, the address was delivered by Dr. W. C. Barrett, of Buffalo, upon the "Diseases of the Period of Dentition." A copy was requested for publication.

The officers elected for the ensuing year are as follows:

*President*—Dr. J. H. Batchelder.

*Vice-President*—Dr. C. P. Wilson.

*Recording Secretary*—Dr. E. E. Hopkins.

*Corresponding Secretary*—Dr. E. B. Hitchcock.

*Treasurer*—Dr. E. H. Smith.

*Librarian*—Dr. H. C. Meriam.

*Executive Committee*—Drs. Chas. Wilson, E. C. Briggs, J. S. Mason.

E. E. HOPKINS, Recording Secretary.

BOSTON, Nov. 5, 1885.

---

### A WANT SUPPLIED.

Although tooth brushes of every conceivable design for brushing the natural organs are everywhere to be found, no one, until recently, has furnished anything suitable for artificial dentures. The Florence Brush Company have now provided just the needed thing. As it is a somewhat delicate matter for wearers of artificial teeth to ask for such brushes at variety shops, why would it not be well for dentists to keep a supply, and furnish to such of their patients as need them.

C. E. F.

---

### MASSACHUSETTS DENTAL SOCIETY.

The next annual meeting of the Massachusetts Dental Society will be held on Thursday and Friday, Dec. 10 and 11, at No. 160 Tremont Street, Boston. Drs. W. H. Atkinson and J. N. Farrar, of New York, will present matters of interest to the profession; also a paper by a prominent medical specialist of this city. Other members of the profession will contribute.

